# SARS-CoV-2 infection, vaccination, and antibody response trajectories in adults: a cohort study in Catalonia

**DOI:** 10.1186/s12916-022-02547-2

**Published:** 2022-09-16

**Authors:** Marianna Karachaliou, Gemma Moncunill, Ana Espinosa, Gemma Castaño-Vinyals, Rocío Rubio, Marta Vidal, Alfons Jiménez, Esther Prados, Anna Carreras, Beatriz Cortés, Natàlia Blay, Marc Bañuls, Vanessa Pleguezuelos, Natalia Rodrigo Melero, Pau Serra, Daniel Parras, Luis Izquierdo, Pere Santamaría, Carlo Carolis, Kyriaki Papantoniou, Ximena Goldberg, Ruth Aguilar, Judith Garcia-Aymerich, Rafael de Cid, Manolis Kogevinas, Carlota Dobaño

**Affiliations:** 1 Barcelona Institute for Global Health (ISGlobal), Doctor Aiguader, 88, 08003 Barcelona, Spain; 2 Centro de Investigación Biomédica en Red de Enfermedades Infecciosas (CIBERINFEC), Barcelona, Spain; 3 Centro de Investigación Biomédica en Red de Epidemiología y Salud Pública (CIBERESP), 08036 Madrid, Spain; 4 Universitat Pompeu Fabra (UPF), Barcelona, Spain; 5 Hospital del Mar Medical Research Institute (IMIM), 08003 Barcelona, Spain; 6 Genomes for Life-GCAT lab. Institute for Health Science Research Germans Trias i Pujol (IGTP), Badalona, Spain; 7 Banc de Sang i Teixits (BST), Barcelona, Spain; 8 Centre for Genomic Regulation (CRG), Barcelona, Spain; 9 Institut d’Investigacions Biomèdiques August Pi Sunyer (IDIBAPS), Barcelona, Spain; 10 Department of Microbiology, Immunology and Infectious Diseases, Snyder Institute for Chronic Diseases, Cumming School of Medicine, University of Calgary, Calgary, Alberta Canada; 11 Department of Epidemiology, Center for Public Health, Medical University of Vienna, Vienna, Austria; 12 Barcelona Institute for Global Health (ISGlobal), Carrer Rosello 132, 08036 Barcelona, Spain

**Keywords:** COVID-19 vaccines, SARS-CoV-2, Antibody, Kinetics, Determinants, Adults, Smoking, Comorbidities

## Abstract

**Background:**

Heterogeneity of the population in relation to infection, COVID-19 vaccination, and host characteristics is likely reflected in the underlying SARS-CoV-2 antibody responses.

**Methods:**

We measured IgM, IgA, and IgG levels against SARS-CoV-2 spike and nucleocapsid antigens in 1076 adults of a cohort study in Catalonia between June and November 2020 and a second time between May and July 2021. Questionnaire data and electronic health records on vaccination and COVID-19 testing were available in both periods. Data on several lifestyle, health-related, and sociodemographic characteristics were also available.

**Results:**

Antibody seroreversion occurred in 35.8% of the 64 participants non-vaccinated and infected almost a year ago and was related to asymptomatic infection, age above 60 years, and smoking. Moreover, the analysis on kinetics revealed that among all responses, IgG RBD, IgA RBD, and IgG S2 decreased less within 1 year after infection. Among vaccinated, 2.1% did not present antibodies at the time of testing and approximately 1% had breakthrough infections post-vaccination. In the post-vaccination era, IgM responses and those against nucleoprotein were much less prevalent. In previously infected individuals, vaccination boosted the immune response and there was a slight but statistically significant increase in responses after a 2nd compared to the 1st dose. Infected vaccinated participants had superior antibody levels across time compared to naïve-vaccinated people. mRNA vaccines and, particularly the Spikevax, induced higher antibodies after 1st and 2nd doses compared to Vaxzevria or Janssen COVID-19 vaccines. In multivariable regression analyses, antibody responses after vaccination were predicted by the type of vaccine, infection age, sex, smoking, and mental and cardiovascular diseases.

**Conclusions:**

Our data support that infected people would benefit from vaccination. Results also indicate that hybrid immunity results in superior antibody responses and infection-naïve people would need a booster dose earlier than previously infected people. Mental diseases are associated with less efficient responses to vaccination.

**Supplementary Information:**

The online version contains supplementary material available at 10.1186/s12916-022-02547-2.

## Background

Natural SARS-CoV-2 infection and vaccination against COVID-19 both contribute to building the population’s immunity. Immune responses are multifaceted but single components that are easy to measure such as antibodies are used in epidemiological studies to answer emerging questions such as what is the percentage of the population lacking antibodies, their characteristics, and the longevity of antibody responses induced after infection and/or vaccination.

Most seroepidemiological studies conducted after COVID-19 vaccine administration began have focused on specific groups (e.g., healthcare workers) [1, 2]. A simultaneous comparison of the robustness of antibody responses across different vaccines and schemes in people with different histories of exposure (non-infected, infected with different symptoms) remains largely unexplored. Thus far, evidence shows that mRNA vaccines induced higher antibody affinity and IgG titers than other vaccine types [3, 4]. Apart from IgG anti-spike responses, vaccines may also induce IgM and IgA responses at variable levels targeting specific immunogens that could contribute to protection. For example, a recent longitudinal assessment of healthcare workers vaccinated with Comirnaty identified different patterns of development of anti-spike IgG and IgM from baseline until 3 weeks after the second dose that were related with virus-neutralizing activity [5]. Serum IgA levels after mRNA vaccination were also associated with protection as reflected in the risk of subsequent breakthrough infection [6]. Also, Dobaño et al. showed the induction of antibodies against the specific C-terminal region of nucleocapsid (NCt) early after administering spike-based mRNA vaccines [7]. These multiple humoral immune responses are less characterized aspects of COVID-19 vaccine-related immunity.

A growing number of studies show the benefit of vaccination in previously infected people although there is still uncertainty on the number of doses required and how this could be modified by vaccine type and time [1–3]. Most studies are limited by the poor allocation of infected and naïve people and the lack of data on the clinical and immunological characteristics of a previous infection. Moreover, current commercially available antibody assays were developed before the variants of concerns (VoCs) emerged and whether the use of antibody assays based on the original Wuhan-Hu-1 strain accurately captures infections with other variants should be evaluated in ongoing seroepidemiological studies.

The durability of antibody responses after infection and/or vaccination is less well explored within the general population [8]. Studies of COVID-19 vaccines have shown waning immunity over time (after 2 doses), as measured by primarily decreasing antibody titers and vaccine effectiveness (breakthrough infections) [9, 10]. Waning antibody titers in the months following natural infection has been observed and especially among persons with mild illness who compose the majority of patients with COVID-19 [11, 12]. In addition, there are intrinsic host factors (such as age, sex, comorbidities) and environmental factors (such as smoking, alcohol consumption, air pollution) that may also influence how individuals respond to vaccines and these factors are less well characterized [13].

In this study, we are following up for a second time during the pandemic an adult population in Catalonia using multiplex antibody serology against SARS-CoV-2 in blood samples collected 6 months after the start of the vaccine rollout in Spain and incorporated questionnaire data and information from healthcare registries. We aimed to assess the SARS-CoV-2 seroprevalence in vaccinated and unvaccinated people and the durability of antibody responses induced by infection and vaccination and to identify major determinants of antibody responses induced by vaccination.

## Methods

### Study design

This analysis uses data longitudinally collected during the COVID-19 pandemic among participants of the Genomes for Life (GCAT) cohort in Catalonia. The GCAT cohort includes mainly middle-aged participants who are residents in Catalonia, and their recruitment started in 2014 [14]. Most participants were enrolled from blood donors invited through the Blood and Tissue Bank and are regularly followed up. We contacted GCAT-eligible participants just after the strict first confinement period in 2020 and almost a year later in 2021 after COVID-19 vaccine administration began in Spain. Participants were contacted via email or telephone and asked to respond to a questionnaire (online or via telephone) and provide a blood sample both in the 2020 and 2021 follow-up. Blood sampling in 2021 was offered to all 2020 participants with a seropositive or an undetermined serostatus (response rate, 47.15%) and to a random sample of 2020 seronegative participants (response rate, 44.21%). People were aware of their 2020 serology results. Additionally to the serology and questionnaire data, results on viral detection tests (SARS-CoV-2 polymerase chain reaction (PCR) and rapid antigen-testing) and immunization data were collected from Electronic Health Records of the Epidemiological Surveillance Emergency Service of Catalonia of the Department of Health. The timeline of serology assessments in our population along with the dates of vaccination administration and the positive testing are all presented in Additional file 1: Fig. S1.

### Serology

Blood samples collected at both time points were processed within 24 h of collection and were analyzed at the ISGlobal Immunology laboratory in Barcelona. The levels [median fluorescence intensity (MFI)] of IgG, IgM, and IgA were assessed by high-throughput multiplex quantitative suspension array technology against a panel of 5 SARS-CoV-2 antigens: the spike full-length protein (S) and the receptor-binding domain (RBD) produced at IDIBAPs (both fused with C-terminal 6xHis and StrepTag purification sequences and purified from the supernatant of lentiviral-transduced CHO-S cells cultured under a fed-batch system) [15], the sub-region S2 (purchased from SinoBiological), the nucleocapsid (N) full length (NFL), and the specific NCt region produced at ISGlobal (both expressed in *E. coli* and His tag-purified [16]). In addition, 4 RBDs from different VoCs, produced at CRG (expressed in Expi294 and His tag purified), were used (with RBD amino acid changes from Wuhan-Hu-1 in parentheses): Alpha (N501Y) (UK, September 2020), Beta (K417N, E484K, N501Y) (South Africa, September 2020), Gamma (K417T, E484K, N501Y) (Brazil, December 2020), and Delta (L452R, T478K) (India, December 2020). Αlpha was the major variant circulating in Europe until early Summer 2021 and was then quickly displaced by the Delta variant, whereas Beta and Gamma accounted only for some cases among those samples sequenced by authorities across Europe at that time [17].

Assay performance was previously established as 100% specificity and 95.78% sensitivity for seropositivity 14 days after symptom onset [18]. Antigen-coupled microspheres were added to a 384-well μClear® flat bottom plate (Greiner Bio-One, Frickenhausen, Germany) in multiplex (2000 microspheres per analyte per well) in a volume of 90 μL of Luminex Buffer (1% BSA, 0.05% Tween 20, 0.05% sodium azide in PBS) using a 384-channel Integra Viaflo semi-automatic device (96/384, 384 channel pipette). Hyperimmune pools were used as positive controls prepared at twofold, 8 serial dilutions from 1:12.5. Pre-pandemic samples were used as negative controls to estimate the cut-off of seropositivity. Ten microliters of each dilution of the positive control, negative controls, and test samples (prediluted 1:50 in 96 round-bottom well plates) was added to a 384-well plate using an Assist Plus Integra device with a 12-channel Voyager pipette (final test sample dilution of 1:500 for all isotypes and a second dilution at 1:5000 for IgG to assess response to S proteins in vaccinated subjects avoiding signal saturation). To quantify IgM and IgA, test samples and controls were pre-treated with anti-human IgG (Gullsorb) at 1:10 dilution, to avoid IgG interferences. Technical blanks consisting of Luminex Buffer and microspheres without samples were added in 4 wells to control for non-specific signals. Plates were incubated for 1 h at room temperature in agitation (Titramax 1000) at 900 rpm and protected from light. Then, the plates were washed three times with 200 μL/well of PBS-T (0.05% Tween 20 in PBS), using BioTek 405 TS (384-well format). Twenty-five microliters of goat anti-human IgG-phycoerythrin (PE) (GTIG-001, Moss Bio) diluted 1:400, goat anti-human IgA-PE (GTIA- 001, Moss Bio) 1:200, or goat anti-human IgM-PE (GTIM-001, Moss Bio) 1:200 in Luminex buffer was added to each well and incubated for 30 min as before. Plates were washed and microspheres resuspended with 80 μL of Luminex Buffer, covered with an adhesive film, and sonicated 20 s on a sonicator bath platform, before acquisition on the Flexmap 3D reader. At least 50 microspheres per analyte and per well were acquired, and MFIs were reported for each isotype-antigen combination. Assay positivity cut-offs specific for each isotype-antigen combination were calculated as 10 to the mean plus 3 standard deviations of log_10_-transformed MFI of 128 pre-pandemic controls. Results were defined as undetermined when the MFI levels for a given isotype-antigen combination were between the positivity threshold and an upper limit at 10 to the mean plus 4.5 standard deviations of the log_10_-transformed MFIs of pre-pandemic samples, and no other isotype-antigen combination was above the positivity cut-off. We defined overall serostatus, by isotype and by antigen.

### Vaccination data

During the course of this study, the vaccines approved for use in Spain were the Comirnaty (BNT162b2, mRNA, BioNTech-Pfizer, Mainz, Germany/New York City, United States (US)); the Spikevax (mRNA-1273, Moderna, Cambridge, US); the Vaxzevria (ChAdOx1 nCoV-19, Oxford–AstraZeneca); and the Janssen COVID-19 vaccine (Ad26.COV2.S, Johnson & Johnson–Janssen). The first doses (January 2021) were distributed among some of the most vulnerable groups, including residents and personnel working in retirement homes, front-line healthcare workers, highly dependent people, and seniors aged 80 and older. Subsequently, essential workers and then adults in descending age order were vaccinated. The government restricted vaccination with Vaxzevria to specific age groups (first upper threshold of 55, then 65, and finally was restricted for people 60–69 years old) and offered the Comirnaty or the Spikevax vaccine as 2nd dose for those <60 years old. The vaccines were offered on a voluntary basis to all individuals.

In this study, we used electronic registries to identify the number of doses, date of administration, and trade names of vaccines for each study participant. We defined participants as non-vaccinated, partially vaccinated (when they had received one dose for those vaccines that have a two-dose regimen and had not acquired infection previously), and fully vaccinated (when they had received two doses for those vaccines that have a two-dose regimen or one dose in those previously infected with SARS-CoV-2 [recommended after 6 months following infection] or one dose for vaccines that have a one-dose regimen).

### Infection data

Performance and results on viral detection tests (PCR or antigen test) were either self-reported or identified by the use of the official repository of tests for SARS-CoV-2 of the Epidemiological Surveillance Emergency Service of Catalonia of the Department of Health [19]. We combined two sources of information because each had its own limitations. Our strategy for detection of participants with evidence of prior infection up to the 2021 serology included (i) previous positive viral detection test, (ii) seropositivity in the 2020 sample (pre-vaccination), (iii) seropositivity in the 2021 sample among non-vaccinated, and (iv) seropositivity to N-antigen in 2021 sample of those vaccinated, since the available vaccines do not contain or produce N-antigen.

### Statistics

Descriptive analyses of the study population characteristics and comparisons between groups were conducted. In all analyses, we used antibody levels log_10_ transformed, due to their skewed distribution. Differences in antibody levels by vaccine type or by vaccination and/or infection were examined using one-way ANOVA and pairwise comparisons were performed using the Tukey post hoc test. Previously infected non-vaccinated individuals were classified for each isotype-antigen combination, as sustainers when the ratio of antibody levels between the two visits ≥1 or decayers when the ratio of antibody levels between the two visits <1 following the previous methodology (10,21). In increasers and decayers, linear mixed-effects models with random intercept and random slope were used to evaluate the trend in antibody levels between the two serology time points, we estimated fold change 1 year after infection separately in sustainers and decayers. Generalized additive models were used to explore the shape of the relationship between days since vaccination and antibody levels among vaccinated people stratified by infection, models were done separately for participants with 1 dose or 2 doses at the time of serology. Among vaccinated people, univariable and multivariable stepwise linear regression models were fit to determine the effect of several variables on the antibody levels. We excluded only people vaccinated with the Janssen COVID-19 vaccine because they were few and eligible only for one dose at that time. We evaluated all the determinants in univariable models and also built a final model for each isotype-antigen combination using forward stepwise regression models including all the described determinants considering 0.1 the significance level for addition to the model and 0.2 for removal from the model. We report associations as a fold change with 95% confidence intervals (FC 95% CI) obtained by 10^beta. We considered the following variables as determinants: age (<60 or ≥60 years old), sex (male, female), highest attained educational level (primary or less, secondary, university), area-based socioeconomic status according to the area of residency (in quantiles), current smoking status (smoker, non-smoker), daily alcohol consumption (yes, no), body mass index (BMI) status (obese, overweight, versus normal/underweight), SARS-CoV-2 infection, vaccine type, and self-reported information on chronic diseases (a disease in the last 6 months that required visit to the doctor or medical treatment) including cardiovascular diseases (hypertension, heart attack, angina pectoris), mental health diseases/addictions (yes, no), and immune-related diseases (rheumatoid arthritis, other autoimmune diseases, HIV or other immunodeficiency, asthma). We adjusted all models a priori for time since the last vaccine dose (<1 month, 1–2 months, 2–3 months, 3–4 months, >4 months) and the number of doses (one or two). Analysis was repeated by vaccine type. Participants with missing covariates were excluded from the final analysis models. We performed all statistical analyses using Stata/SE (version 16; StataCorp LLC.).

## Results

### Study population characteristics

Overall, 1076 people were included in this analysis, aged 43–72 years old and 59.1% were female. By the time of sample collection (May 26–July 21, 2021), 130 (12.1%) participants were not vaccinated, 267 (24.8%) were partially vaccinated, and 679 (63.1%) were fully vaccinated according to the guidelines in force at that time (details in the “Methods” section). We detected a heterologous prime-boost approach in 11 people (Vaxzevria as a first shot followed by Comirnaty). Among vaccinated participants, the first dose was either Comirnaty (44.4 %) or Vaxzevria (41.9%) and a smaller fraction of people was vaccinated with Spikevax (12%) or Janssen COVID-19 vaccine (1.7%). Median time since the last vaccination was 28 days (IQR: 14–54 days, min 1 day, max 160 days). The median time between 1st and 2nd doses was 21 days for Comirnaty (IQR: 21–22 days, min 14 days, max 42 days), 28 days for Spikevax (IQR: 28–29 days, min 27 days, max 36 days), and 83 days for Vaxzevria (IQR: 78–95 days, min 63 days, max 121 days). A higher proportion of non-vaccinated participants had a previous positive viral detection test (32.3%) or positive serology in 2020 (49.2%) compared to vaccinated participants (12.5% and 28.2% respectively). Post-vaccination, approximately 1% of the people presented breakthrough infections detected by viral detection tests. Three participants were infected after the first vaccine dose and six after the 2nd vaccine dose (Additional file 1: Table S1).

### SARS-CoV-2 infections and durability of antibody responses

**Fig. 1:**
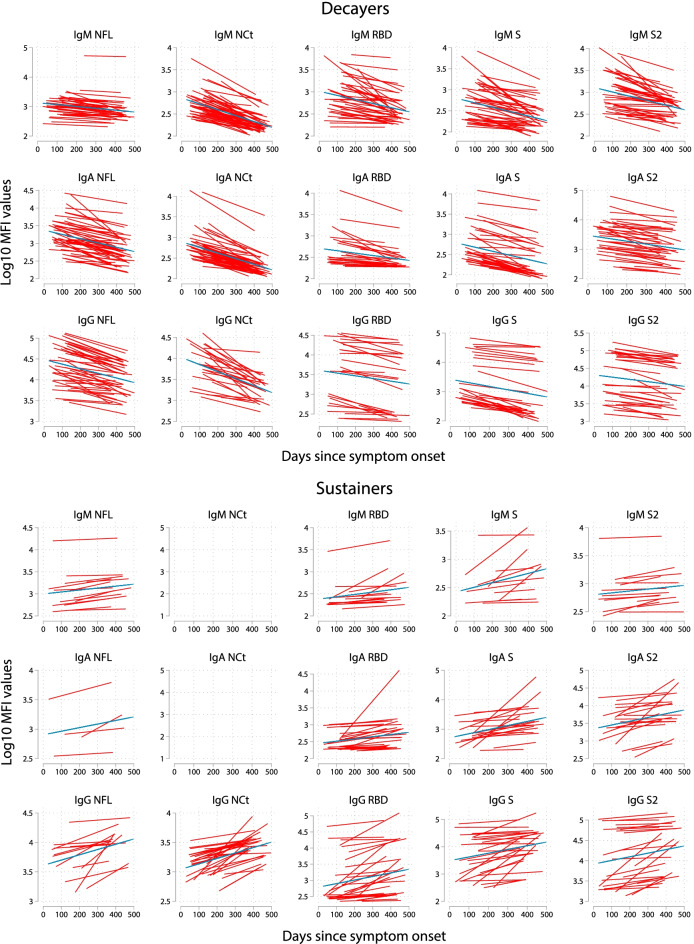
Kinetics of SARS-CoV-2 antibody levels since the onset of symptoms among decayers and sustainers. Decayers and sustainers are specific for each isotype-antigen combination. Antibody levels (median fluorescence intensity, MFI) are log_10_ transformed and are measured twice (paired samples joined by lines). The blue solid line represents the fitted curve calculated using linear mixed-effects models

In our study sample, 395 (36.7%) people had been infected incorporating data from serology in 2020 and 2021, registries, and questionnaires (Additional file 1: Table S2). A total of 64 participants were infected early in the pandemic (spring-summer 2020) and remained non-vaccinated up to their second serological assessment in 2021, with more than 300 days after primary infection. Among them, 43 (67.2%) were still seropositive, 8 (15.5%) had an undetermined status, and 13 (20.3%) were seronegative in the 2021 assessment. Those who seroreverted were more likely to have experienced an asymptomatic infection (61.9% vs 25.6%), be above 60 years of age (42.8% vs 34.8%), and smoke (19% vs 4.6%). Those who remained seropositive were more likely to have had in 2020 higher breadth of immune responses (3.5 vs 0.7, aggregate number of positive isotype-antigen combinations) and be IgA and/or IgG positive against RBD and S in 2020 (e.g., for IgG RBD 60.5% vs 4.8%). Comparing the antibody levels between the two visits, we observed that for each isotype-antigen combination there were sustainers and decayers. We noted that most individuals qualifying as IgG sustainers to S or N antigens also sustained production of IgA specific to S antigens (Additional file 1: Fig. S2). Plotting antibody levels in time since infection showed the trend of stable/increasing antibody levels in sustainers and decreasing antibody levels in decayers (Fig. 1). Among decayers, the highest reductions in responses within 1 year after infection were for IgG NCt (75.4% decrease), IgA NCt (68.1%), IgA NFL (64.1%), and IgG S (63.9%) and the lowest for IgA RBD (38.1%), IgG S2 (42.2%), and IgG RBD (44.2%) (Additional file 1: Table S3).

### Sharp increase in positive spike responses after vaccine rollout

**Fig. 2:**
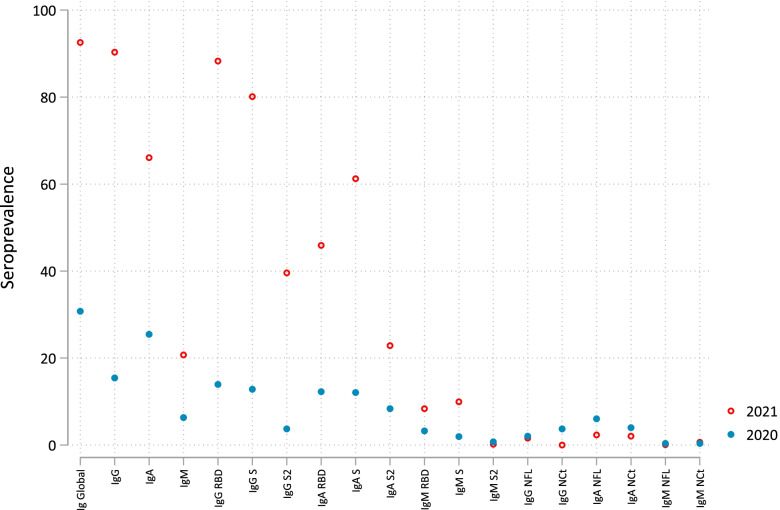
SARS-CoV-2 overall seroprevalence and by isotype and isotype-antigen combination in 1076 adults followed up in 2020 and 2021

From 2020 to 2021 serological assessment (duration ranging from 6 to 13 months), the overall seroprevalence of our study sample increased from 30.8 to 92.6% (Fig. 2). The most remarkable changes in isotype-antigen combinations were for IgG and IgA responses against RBD and S, e.g., RBD IgG seropositivity increased from 13.9 to 88.3% as expected after vaccination. Seroprevalence increased significantly for all responses except for responses against NFL and NCt and IgM-S2 that were reduced or remained unchanged.

We should note that we based the overall serostatus in 2021 only on RBD Wuhan responses. This is because Spearman correlation between RBD log_10_-transformed responses of different VoCs was generally high (Additional file 1: Table S4). Moreover, among individuals who tested negative/undetermined for antibodies to RBD Wuhan, five were RBD positive for at least another VoC (Additional file 1: Table S5). Among RBD Wuhan seropositive, there were 215 people with no detectable antibodies against RBD to at least one other VoC and 34 people with no detectable antibodies to the RBD of all other VoCs.

### Antibody responses by vaccination status

Table 1 presents the SARS-CoV-2 seroprevalence in 2021 according to the vaccination status of the individuals. Among those vaccinated, almost all were seropositive (99.3% of fully and 94.4% of partially vaccinated). Twenty vaccinated people (2.1%) did not present antibodies at the time of testing (seronegative or with an undetermined status). Among them, there were nine people who received the 1st dose of Comirnaty within the last 7 days, five who received the 1st dose of Vaxzevria more than 65 days ago (all >60 years of age, one female), four who received the 2nd dose of Vaxzevria the last 5 days, and one person with Janssen COVID-19 vaccination (1 dose) in the last 9 days (Additional file 1: Table S6). Vaccinated individuals presented IgG but also IgA and to a lesser extent IgM-positive responses. For all isotypes, positive responses were more prevalent in vaccinated vs non-vaccinated people. N-positive responses were more prevalent in non-vaccinated people whereas spike-positive responses in vaccinated participants. IgG RBD responses after vaccination were comparable between Wuhan, Alpha, and Delta variants but lower for Beta and Gamma variants (Additional file 1: Fig. S3).

**Table 1:** Overall SARS-CoV-2 seroprevalence (values for seronegativity are not included) by isotype and isotype-antigen combination in the study participants by vaccination status (*n* = 1.076)

	Fully vaccinated		Partially vaccinated		Non-vaccinated	
	Positive	Undetermined	Positive	Undetermined	Positive	Undetermined
	*n* (%)	*n* (%)	*n* (%)	*n* (%)	*n* (%)	*n* (%)
Overall	674 (99.3)	3 (0.4)	252 (94.4)	5 (1.9)	70 (53.8)	13 (10.0)
By isotype						
IgM	160 (23.6)	88 (13.0)	46 (17.2)	26 (9.7)	17 (13.1)	13 (10.0)
IgA	509 (75.0)	56 (8.2)	148 (55.4)	29 (10.9)	54 (41.5)	11 (8.5)
IgG	673 (99.1)	3 (0.4)	240 (89.9)	7 (2.6)	59 (45.4)	8 (6.2)
By antigen						
N	33 (4.9)	66 (9.7)	28 (10.5)	20 (7.5)	18 (13.8)	18 (13.8)
NFL	21 (3.1)	59 (8.7)	16 (6.0)	27 (10.1)	15 (11.5)	17 (13.1)
NCt	13 (1.9)	26 (3.8)	14 (5.2)	6 (2.2)	6 (4.6)	3 (2.3)
RBD	670 (98.7)	5 (0.7)	231 (86.5)	12 (4.5)	60 (46.2)	7 (5.4)
S	655 (96.5)	19 (2.8)	208 (77.9)	40 (15.0)	58 (44.6)	11 (8.5)
S2	449 (66.1)	171 (25.2)	111 (41.6)	70 (26.2)	47 (36.2)	18 (13.8)
By isotype-antigen combination						
IgM NFL	0 (0.0)	5 (0.7)	1 (0.4)	8 (3.0)	0 (0.0)	3 (2.3)
IgM NCt	2 (0.3)	10 (1.5)	5 (1.9)	1 (0.4)	0 (0.0)	1 (0.8)
IgM RBD	60 (8.8)	87 (12.8)	19 (7.1)	27 (10.1)	11 (8.5)	10 (7.7)
IgM S	87 (12.8)	137 (20.2)	15 (5.6)	37 (13.9)	5 (3.8)	11 (8.5)
IgM S2	1 (0.1)	18 (2.7)	0 (0.0)	7 (2.6)	1 (0.8)	2 (1.5)
IgA NFL	14 (2.1)	27 (4.0)	9 (3.4)	18 (6.7)	2 (1.5)	12 (9.2)
IgA NCt	9 (1.3)	17 (2.5)	8 (3.0)	7 (2.6)	5 (3.8)	2 (1.5)
IgA RBD	382 (56.3)	64 (9.4)	82 (30.7)	13 (4.9)	30 (23.1)	9 (6.9)
IgA S	490 (72.2)	57 (8.4)	129 (48.3)	21 (7.9)	40 (30.8)	16 (12.3)
IgA S2	175 (25.8)	217 (32.0)	52 (19.5)	68 (25.5)	19 (14.6)	29 (22.3)
IgG NFL	4 (0.6)	42 (6.2)	2 (0.7)	13 (4.9)	11 (8.5)	12 (9.2)
IgG NCt	0 (0.0)	3 (0.4)	0 (0.0)	0 (0.0)	0 (0.0)	2 (1.5)
IgG RBD	668 (98.4)	6 (0.9)	224 (83.9)	13 (4.9)	58 (44.6)	4 (3.1)
IgG S	647 (95.3)	26 (3.8)	175 (65.5)	64 (24.0)	40 (30.8)	21 (16.2)
IgG S2	344 (50.7)	264 (38.9)	52 (19.5)	103 (38.6)	30 (23.1)	27 (20.8)

All *p*-values <0.05 except for IgM RBD, IgM S2, IgA NFL, IgA NCt, and IgG NCt

**Fig. 3:**
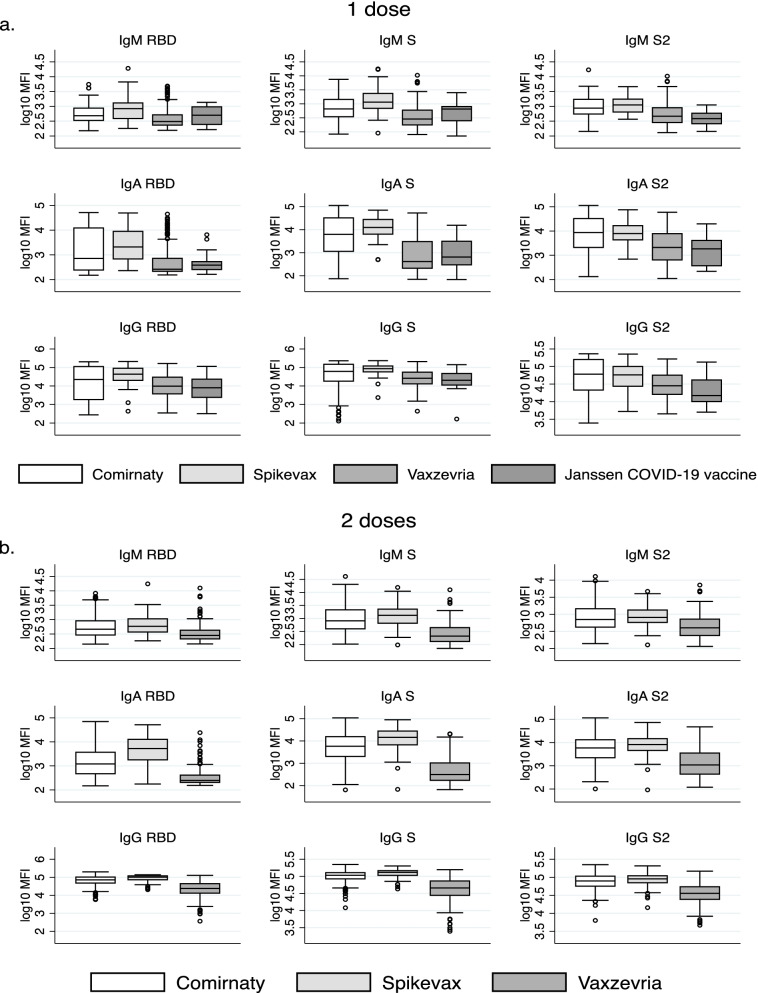
Differences in antibody responses by vaccine type among those who have received **a** one dose and **b** two doses. Additional file 1: Table S8 presents corresponding *p*-values

People vaccinated with mRNA vaccines (Comirnaty or Spikevax) had significantly higher levels of IgM, IgA, and IgG against RBD, S, and S2 compared to Vaxzevria or Janssen COVID-19 vaccination (Fig. 3a, b). The pattern was consistent after 1st and 2nd doses. Spikevax compared to Comirnaty vaccination was associated to higher IgG and IgA responses, although after 1 dose, differences were only found for IgG against RBD and S2. Also, IgM S levels after 1st dose were statistically significantly higher in people vaccinated with Spikevax compared to Comirnaty (Fig. 3a).

### Antibody trajectories up to 5 months after vaccination (1st and 2nd doses)

**Fig. 4:**
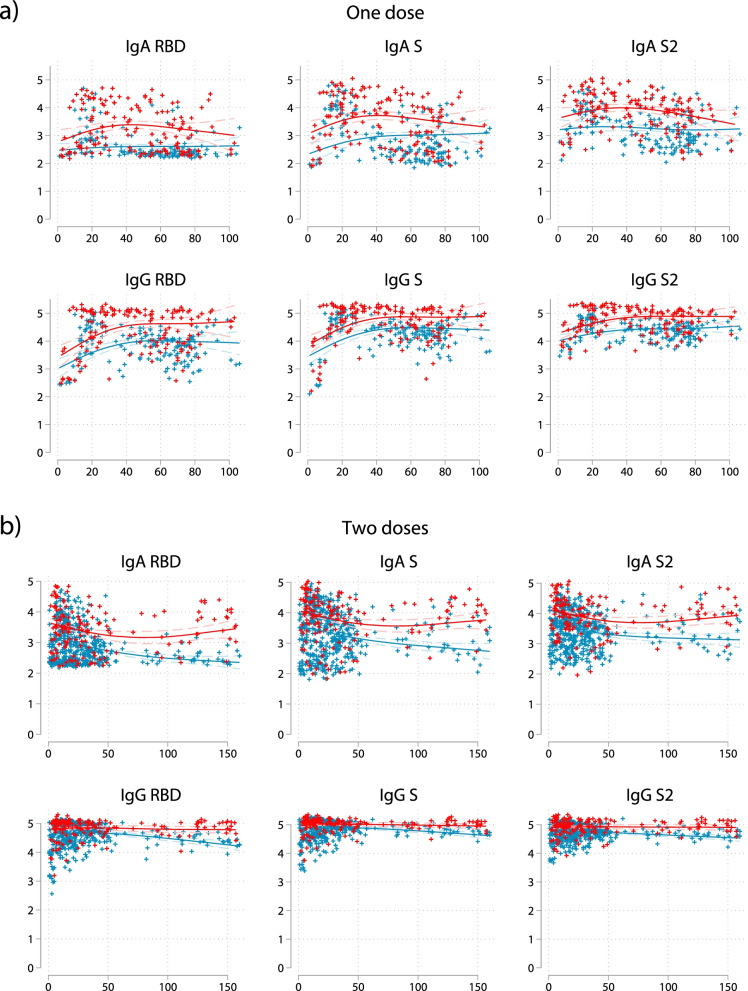
Generalized additive models for associations of days since vaccination with antibody responses to the six isotype-antigen combinations in infected (red) and naïve (blue) participants after the **a** first and **b** second doses. Fitted lines after adjustment for participant’s age and type of vaccine. Plus symbols (+) represent measured responses for a specific participant. People vaccinated with the Janssen COVID-19 vaccine are not considered in this analysis

We examined the effect of time since vaccination on antibody levels against RBD, S, and S2 using cross-sectional data from all vaccinated participants with time since the last vaccination ranging from 1 to 160 days, adjusting for participant’s age and vaccine type and stratified by infection (Fig. 4a, b). Previously infected people mounted higher antibody levels after the 1st and 2nd doses than naive individuals. Differences between infected and naïve people were larger in IgA than IgG responses. After the first dose, IgA and IgG responses peaked between 20 and 40 days after vaccination, although in naïve individuals, IgA responses against RBD and S2 remained unchanged in time. In infected people, after the 2nd dose, IgA responses appeared to be highest just after the shot, decayed rapidly, and then stabilized at around 50 days or slightly increased between 100 and 150 days (reverse J-shape). In naïve individuals, IgA responses decayed just after the 2nd dose. IgG responses in infected people with two doses remained unchanged in time up to 5 months, whereas in naïve people there was a slight decay with time after 50 days since 2nd dose. Corresponding plots by vaccine type are presented in Additional file 1: Fig. S4.

### Antibody responses after vaccination in SARS-CoV-2-infected and naïve individuals

**Fig. 5:**
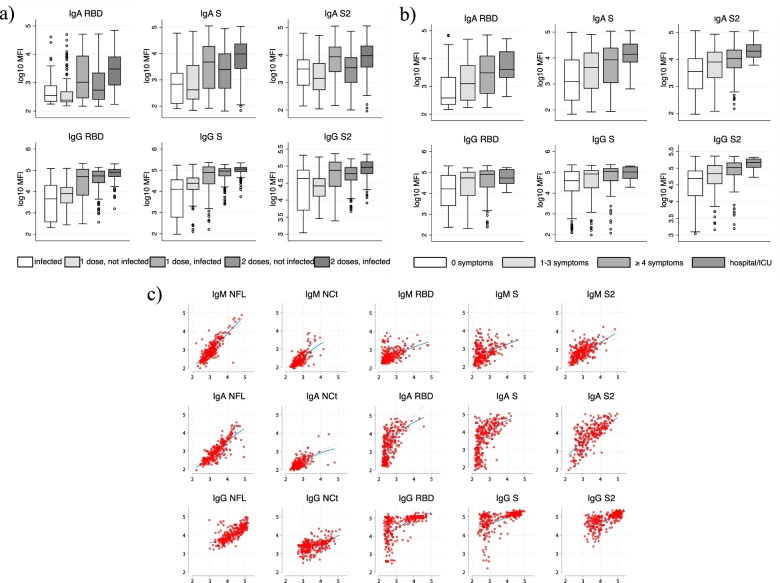
**a** Differences in antibody responses by infection and/or vaccination and number of doses. **b** Differences in antibody responses after vaccination in previously infected participants by the severity of infection. **c** Association between antibody levels induced after infection and antibody levels after vaccination adjusting for age (continuous), gender, type of vaccine, number of doses, and time since last vaccination. People vaccinated with the Janssen COVID-19 vaccine are not considered in this analysis. Additional file 1: Table S8 presents corresponding *p*-values

Figure 5a shows antibody levels to different isotype-antigen combinations by infection and/or vaccination status (vaccinated with Janssen COVID-19 vaccine not included). In infected people, the first vaccination significantly boosted IgA and IgG responses to RBD, S, and S2 compared to those non-vaccinated. A 2nd dose significantly increased IgA RBD, S and IgG RBD, S, and S2 compared to the 1st dose — especially in infected people vaccinated with Comirnaty, although the increase was small (Additional file 1: Fig. S5). No statistically significant differences were observed between 1st and 2nd doses for infected people vaccinated with Spikevax or Vaxzevria. For all vaccine types, 2nd vs 1st dose was associated with higher IgG and IgA levels among non-infected people. Moreover, IgM S levels were highest in people receiving 2 doses (Additional file 1: Fig. S6). IgA and IgG responses to NFL and NCt were higher among those infected ± vaccinated than those vaccinated with no evidence of infection (Additional file 1: Fig. S5). In infected people, the clinical and immunological characteristics were strongly related to the antibody responses after vaccination. In detail, people who had experienced a more severe infection presented higher antibody levels after vaccination, with the highest levels being among those hospitalized (Fig. 5b). Moreover, there was a positive strong association between antibody levels induced by infection and antibody levels following vaccination (Fig. 5c). This analysis was performed among 262 vaccinated participants who were tested seropositive in 2020 and models were adjusted for age, sex, type of vaccine, number of doses, and time since the last vaccination.

### Determinants of antibody levels induced after vaccination

**Fig. 6:**
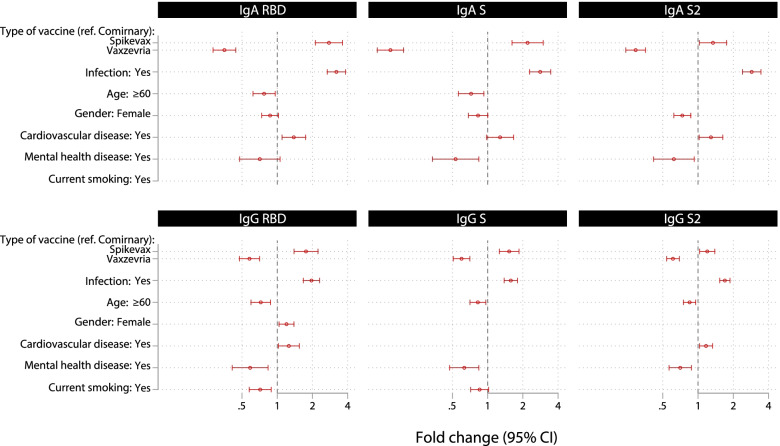
Forest plot showing fold change and 95% confidence intervals for antibody responses versus covariates selected from stepwise linear regression models, among participants who had received at least one vaccine dose. Final models presented here are specific for each isotype-antigen combination and are all a priori adjusted for time since the last vaccine dose and number of doses. Univariate associations are presented in Additional file 1: Table S7

In stepwise multivariable regression models (Fig. 6), the vaccine type was the major modifiable determinant of all antibody responses. Specifically, compared to Comirnaty vaccination Vaxzevria was negatively associated with most IgA and IgG responses whereas Spikevax was positively associated. Smoking was significantly associated with lower RBD IgG levels. SARS-CoV-2 infected vs non-infected had higher IgA and IgG levels. People with cardiovascular disease presented higher IgA and IgG RBD and S2 levels. Older age (≥60 years old) and mental disease were negatively associated with most IgA and IgG responses. Among other potential determinants explored in univariable models, obesity was negatively associated with IgG RBD and S responses but was not selected in the stepwise approach (Additional file 1: Table S7).

## Discussion

Our analysis, based on a middle-aged adult population followed up twice during the pandemic with measures of multiple antibody responses against SARS-CoV-2 before and after vaccination, identified key aspects in both infection-acquired and vaccine-acquired immunity. First, 35.8% of infected non-vaccinated participants had no longer detectable antibodies over a year after infection. Second, vaccination induced higher responses in previously infected individuals and they had considerably higher antibody levels over time compared to naïve-vaccinated people. Third, clinical phenotype and antibody levels after infection were strongly associated with the antibody responses after vaccination. Fourth, vaccine type was the major modifiable factor of antibody responses after vaccination and Spikevax resulted in the highest responses. Other intrinsic and extrinsic factors also determined the vaccination responses.

A very small (7.5%) proportion of our population had no detectable antibodies in the 2021 serological assessment and was likely susceptible to COVID-19. These people are a target group for vaccination campaigns. Most of them were not vaccinated people and around half of those reported a previous infection. Studies on vaccine hesitancy highlight that having experienced an infection is one of the reasons for not being vaccinated [20, 21]. We showed that 35.8% of people infected almost a year ago and not vaccinated were no longer seropositive particularly if they were >60 years of age, were smokers, and had experienced an asymptomatic infection. Other studies have shown a similar [22] or lower (e.g., 4% in healthcare workers) [23] proportion of people with no longer detectable antibodies a year after infection. We have shown that infected people may benefit from vaccination given the significant increase in antibody levels after vaccination, in line with other reports [2, 24, 25]. Beyond that, there is emerging evidence on the protective role of vaccination on post-acute COVID-19 symptoms [26–28].

Higher IgA and IgG responses against S antigens were produced among infected vaccinated people compared to their naïve-vaccinated peers. This difference in responses was shown to persist 5 months after vaccination. Bates et al. showed that a boost in humoral immune responses occurs regardless of whether the infection is acquired before or after any vaccination and concerns neutralizing and binding antibodies as well [29]. In our population, almost 40% of the vaccinated people had evidence of a previous infection implying that an important proportion has superior immune responses due to hybrid immunity. Further research is warranted to understand the interplay between natural and vaccine-induced immunity and the role of exposure to specific variants and the interval between exposures (vaccination doses and infection). The need for further booster doses should take into consideration both vaccine doses and infections and also the clinical characteristics during infections. People who have experienced a more severe infection and had higher antibody levels after infection had also higher levels after vaccination, similar to results from a study among healthcare workers [2]. Thus, the way people responded after the first exposure to SARS-CoV-2 was associated with the way they responded after vaccination. Research in other infectious diseases supports that previous or even first exposure shapes our immune system and this might have important implications in improving vaccine-induced immunity [30].

Only a few vaccinated people had no detectable antibodies at the time of sampling and this was related to the vaccine type and the time since vaccination uptake (either too close to the 1st shot of Comirnaty or too far to the 2nd dose of Vaxzevria). Vaccine type among other characteristics was one of the determinants of all antibody responses after vaccination. We confirmed that mRNA vaccination was associated with higher antibody levels compared to other vaccine types [2]. This was reflected in IgG but also IgA and IgM levels and probably explains the highest efficacy of these vaccines [31]. Particularly, Spikevax presented the highest responses among all. Lafon et al. also confirmed higher IgA and IgG titers in people vaccinated with mRNA vs vector-based COVID-19 vaccines [32]. It is suggested that a coordinated humoral response is associated with increased protective immunity [33]. Previous studies have highlighted the role of IgA in virus neutralization early in the infection [34] and that high anti-spike IgA serum levels post-vaccination are associated with protection against subsequent breakthrough infection [6]. Far less data are available for IgM-specific responses and their association with vaccine efficacy [5]. Future follow-up of our population will allow us to assess the risk and severity of breakthrough infections in people with different vaccine responses covering also dynamic aspects of multiple isotype and antigen responses. Moreover, infected people receiving two doses of Spikevax had higher levels compared to those receiving only one dose. This difference was not significant in other vaccine types. In the COV-BOOST trial, assessing the comparative safety and immunogenicity of different COVID-19 vaccines given as a 3rd (booster) dose, Spikevax vaccine produced the biggest antibody response among seven vaccines [35].

We found that individuals 60 years or older had lower antibody levels after vaccination compared to younger participants and increased age has been associated with reduced vaccine responses in a number of studies irrespective of vaccine type [36, 37]. A similar effect has been seen with antibody responses after infection [11]. Across a number of studies, women had higher IgG levels after COVID-19 vaccination than men, a finding also observed in our study [1, 38]. No previous studies have reported sex differences in IgA responses highlighting the need to investigate sex differences for a wider number of immune responses. Sex-specific responses are also evident in a range of other vaccines (e.g., females have higher antibodies after hepatitis B while males after tetanus vaccination) [13].

Smokers had considerably lower antibody levels after vaccination and infected smokers were more likely to serorevert a year after infection. A recent systematic review indicated that active smoking negatively impacts humoral response to COVID-19 vaccines but there is limited evidence on the underlying pathophysiologic mechanisms [39]. People reporting mental health disease presented lower antibody levels in multivariable models. To our knowledge, studies examining the effect of mental illness on COVID-19 vaccination response are scarce but in line with our findings [1]. This is an area of further research because associations may be attributable to specific diagnoses, medications, and other underlying characteristics of those people such as sleep disturbances. Nonetheless, mental illness is associated with altered immune function and evidence shows a reduced immune response to vaccination for people with depression, chronic stress, schizophrenia, bipolar disorder, etc. [40]. Interestingly, people with cardiovascular disease mounted higher IgA and IgG responses after vaccination. Of note, these people did not report more frequently persistent chest pain or tightness or cardiovascular sequelae. Results from other studies are mixed on this topic [1, 41]. Obesity was associated with lower vaccine responses in the univariable models only. Although a number of studies link obesity with lower antibody responses after COVID-19 vaccines [1] and non-COVID-19 vaccines, some studies fail to find an association [42].

This study was limited by its use of a sample nested within a cohort mainly of blood donors, which may limit the generalizability of the results. Focus on early time periods after vaccination limits our analysis on the durability of responses induced by vaccines. Testing people at a later phase of the vaccination campaign would probably result in more people fully vaccinated but we were able to show the effect of different combinations of the number of doses and type of vaccine on antibody levels. Another issue is that we measured binding but not neutralizing antibodies, which is arguably a better surrogate of immune protection. IgG levels to S and RBD and neutralization titers are typically highly correlated [15]. Another limitation is that only a low number of vaccinated individuals received the Janssen COVID-19 vaccine, limiting our ability to describe responses related to this vaccine. This study is strengthened by its large sample size and detailed serological assessment twice over the course of the pandemic. Combining appropriate information from serology, questionnaires, and registries, we were able to identify previously infected people as accurately as possible and avoid many sources of bias that would lead to differential misclassification of people as infected. Finally, both serological assessments were performed in the same lab using identical assays and technologies.

## Conclusions

In summary, in this large cohort study, we compared the antibody responses induced by different COVID-19 vaccines, in people with different histories of exposure to the virus and underlying characteristics. All these differences accounted for non-homologous vaccine responses among individuals. We showed that infection and vaccination resulted in higher and longer-lasting antibody responses compared to vaccination alone, in the long term as well. Importantly, the response to primary infection was strongly related to the response to vaccination, adding to the growing literature on the potency of previous (or first) exposures to shape our immune system. Moreover, in addition to the well-studied determinants of antibody responses after vaccination (e.g., age, sex, smoking), we identified other novel determinants (e.g., cardiovascular and mental health diseases). Taken together, our results indicate that vaccination campaigns should be tailored according to vaccines available, previous history of SARS-CoV-2 infection, and characteristics of the population in order to achieve optimal responses and protection as possible across individuals.

## Supplementary Information

**Additional file 1: Table S1**. Characteristics of participants with breakthrough infections, post-vaccination. **Table S2**. Allocation of vaccinated and non-vaccinated participants with evidence of infection according to the criterion of infection fulfilled. **Table S3**. Fold change (FC) (95% CI) in antibody levels within one year after infection estimated using two repeated samples among decayers. Estimates are based on linear mixed-effects models. **Table S4**. Spearman correlations for RBD antigen. All participants, *n*=1,076. Darker red=stronger association. **Table S5**. Cross tabulation between serostatus to RBD of Wuhan variant with the RBD of Alpha, Beta Gamma and Delta variants. **Table S6**. Characteristics of non-responders (seronagetive or with an undetermined status) to vaccination. **Table S7**. Association (fold change FC and 95% CI and *p*-values) between each determinant with log10 antibody leves in vaccinated people after adjusting each model for time since last vaccination and number of doses. Participants with any vaccination excluding Janssen (*n*=923). **Table S8**. *P*-values for comparisons related to Figs. 3 and 5 and Figure S6. **Figure S1**. Dates and density of positive viral detection tests, sampling in 2020 (1st serological assessment) and 2021 (2nd serological assessment) and receipt of 1st vaccine dose in the study population (*n*=1,076). **Figure S2**. Venn diagram illustrating overlap between sustainer groups of IgA or IgG antibodies against nucleoprotein and spike antigens, among all infected unvaccinated participants (*n*=64). **Figure S3**. Differences in IgG antibody responses against RBD between Wuhan, Alpha, Beta, Gamma and Delta variant among vaccinated people. All differences were statistically significant apart from Delta vs Wuhan (*p*=0.861) and Alpha vs Wuhan (*p*=0.051). **Figure S4**. Generalized additive models for associations of days since vaccination with antibody responses to the six isotype-antigen combinations in infected (red) and naïve (blue) participants after first or second dose in people vaccinated by Vaxzevria (a), Comirnaty (b) or Spikevax (c). Fitted lines after adjustment for participant’s age. Plus symbols (+) represent measured responses for a specific participant. **Figure S5**. Differences in antibody responses by infection and/or vaccination and number of doses in people vaccinated with Comirnaty (a), Spikevarx (b), Vaxzevria (c) of Janssen COVID-19 vaccine (d). **Figure S6**. Differences in IgM responses by infection and/or vaccination and number of doses. **Table S8** presents corresponding *p*-values.

## Data Availability

The datasets used and/or analyzed during the current study are available from the corresponding author on reasonable request. Protocol information will be available on reasonable request.

## Abbreviations

BMI: Body mass index
COVID-19: Coronavirus disease 2019
IgA: Immunoglobulin A
IgG: Immunoglobulin G
IgM: Immunoglobulin M
MFI: Median fluorescence intensity
NCt: Nucleocapsid C-terminal region
NFL: Nucleocapsid full protein
PCR: Polymerase chain reaction
RBD: Receptor-binding domain
S: Spike full protein
S2: S2 fragment
SARS-CoV-2: Severe acute respiratory syndrome coronavirus 2
VoCs: Variants of concerns
