# Supplementary material for: SARS-CoV-2 infection, vaccination, and antibody response trajectories in adults: a cohort study in Catalonia
Source: BMC Med. 2022 Sep 16;20:347. doi: 10.1186/s12916-022-02547-2 (PMC9479347; doi:10.1186/s12916-022-02547-2)
Supplement: Supplementary file 1 — Additional file 1: Table S1. Characteristics of participants with breakthrough infections, post-vaccination. Table S2. Allocation of vaccinated and non-vaccinated participants with evidence of infection according to the criterion of infection fulfilled. Table S3. Fold change (FC) (95% CI) in antibody levels within one year after infection estimated using two repeated samples among decayers. Estimates are based on linear mixed-effects models. Table S4. Spearman correlations for RBD antigen. All participants, n=1,076. Darker red=stronger association. Table S5. Cross tabulation between serostatus to RBD of Wuhan variant with the RBD of Alpha, Beta Gamma and Delta variants. Table S6. Characteristics of non-responders (seronagetive or with an undetermined status) to vaccination. Table S7. Association (fold change FC and 95% CI and p-values) between each determinant with log10 antibody leves in vaccinated people after adjusting each model for time since last vaccination and number of doses. Participants with any vaccination excluding Janssen (n=923). Table S8. P-values for comparisons related to Figs. 3 and 5 and Figure S6. Figure S1. Dates and density of positive viral detection tests, sampling in 2020 (1st serological assessment) and 2021 (2nd serological assessment) and receipt of 1st vaccine dose in the study population (n=1,076). Figure S2. Venn diagram illustrating overlap between sustainer groups of IgA or IgG antibodies against nucleoprotein and spike antigens, among all infected unvaccinated participants (n=64). Figure S3. Differences in IgG antibody responses against RBD between Wuhan, Alpha, Beta, Gamma and Delta variant among vaccinated people. All differences were statistically significant apart from Delta vs Wuhan (p=0.861) and Alpha vs Wuhan (p=0.051). Figure S4. Generalized additive models for associations of days since vaccination with antibody responses to the six isotype-antigen combinations in infected (red) and naïve (blue) participants after first or [file 12916_2022_2547_MOESM1_ESM.docx]

**Additional file 1**

**SARS-CoV-2 infection, vaccination and antibody response trajectories in adults: a cohort study in Catalonia**

**Authors**

Marianna Karachaliou, Gemma Moncunill, Ana Espinosa, Gemma Castaño-Vinyals, Rocío Rubio, Marta Vidal, Alfons Jiménez, Esther Prados, Anna Carreras, Beatriz Cortés, Natàlia Blay, Marc Bañuls, Vanessa Pleguezuelos, Natalia Rodrigo Melero, Pau Serra, Daniel Parras, Luis Izquierdo, Pere Santamaría, Carlo Carolis, Kyriaki Papantoniou, Ximena Goldberg, Ruth Aguilar, Judith Garcia-Aymerich, Rafael de Cid, Manolis Kogevinas, Carlota Dobaño

**Supplementary Tables**

| Table S1. Characteristics of participants with breakthrough infections, post-vaccination. | | | | | | | |
| --- | --- | --- | --- | --- | --- | --- | --- |
| age | gender | 1^st^ vaccine dose | | 2^nd^ vaccine dose | | Date of infection, post-vaccination | Date of infection, pre-vaccination |
|  |  | type | date | type | date |  |  |
| 66 | Male | Vaxzevria | 27-Mar-21 | no |  | 07-May-21 |  |
| 61 | Male | Comirnaty | 13-Jan-21 | Comirnaty | 10-Feb-21 | 24-Jan-21 | 03-Apr-20 |
| 50 | Female | Comirnaty | 11-Feb-21 | Comirnaty | 05-Mar-21 | 23-Feb-21 | 25-Mar-20 |
| 54 | Male | Comirnaty | 18-Jan-21 | Comirnaty | 11-Feb-21 | 16-Feb-21 |  |
| 45 | Female | Comirnaty | 12-Feb-21 | Comirnaty | 05-Mar-21 | 30-Mar-21 | 20-May-20 |
| 55 | Female | Comirnaty | 11-May-21 | Comirnaty | 02-Jun-21 | 08-Jul-21 |  |
| 50 | Female | Spikevax | 23-May-21 | Spikevax | 21-Jun-21 | 09-Jul-21 | 30-Mar-20 |
| 47 | Female | Comirnaty | 05-Jan-21 | Comirnaty | 26-Jan-21 | 02-Feb-21 | 30-Apr-20 |
| 52 | Male | Comirnaty | 12-Jan-21 | Comirnaty | 03-Feb-21 | 20-Jun-21 | 13-May-20 |

|  | | | | | |
| --- | --- | --- | --- | --- | --- |
| Table S2. Allocation of vaccinated and non-vaccinated participants with evidence of infection according to the criterion of infection fulfilled. | | | | |  |
|  | Evidence of infection | | | |  |
|  | previous positive viral detection test | seropositivity in 2020 samples (pre-vaccination) | seropositivity to N antigen in 2021 samples among vaccinated | seropositivity in 2021 samples among non vaccinated |  |
| vaccinated |  |  |  |  |  |
| n=25 | yes |  |  |  |  |
| n=72 | yes | yes |  |  |  |
| n=3 | yes |  | yes |  |  |
| n=143 |  | yes |  |  |  |
| n=34 |  | yes | yes |  |  |
| n=6 |  |  | yes |  |  |
| n=18 | yes | yes | yes |  |  |
| non-vaccinated |  |  |  |  |  |
| n=6 |  |  |  | yes |  |
| n=27 |  | yes |  | yes |  |
| n=16 | yes | yes |  | yes |  |
| n=21 | yes |  |  | yes |  |
| n=3 | yes |  |  |  |  |
| n=2 | yes | yes |  |  |  |
| n=19 |  | yes |  |  |  |

| Table S3. Fold change (FC) (95% CI) in antibody levels within one year after infection estimated using two repeated samples among decayers. Estimates are based on linear mixed-effects models | | | |
| --- | --- | --- | --- |
|  | FC (95%CI) | % change (95% CI) | n |
| IgM NFL | 0,59 (0,52, 0,66) | -41,2 (-47,7, -33,8) | 44 |
| IgM NCt | 0,33 (0,29, 0,37) | -67,4 (-70,9, -63,4) | 58 |
| IgM RBD | 0,46 (0,36, 0,57) | -54,4 (-63,6, -42,9) | 43 |
| IgM S | 0,41 (0,33, 0,50) | -59,1 (-66,7, -49,7) | 46 |
| IgM S2 | 0,42 (0,34, 0,52) | -57,5 (-65,6, -47,6) | 44 |
| IgA NFL | 0,36 (0,30, 0,43) | -64,1 (-69,8, -57,2) | 54 |
| IgA NCt | 0,32 (0,28, 0,36) | -68,1 (-71,9, -63,8) | 58 |
| IgA RBD | 0,62 (0,52, 0,74) | -38,1 (-47,9, -26,4) | 30 |
| IgA S | 0,42 (0,36, 0,48) | -58,1 (-63,8, -51,5) | 38 |
| IgA S2 | 0,44 (0,37, 0,51) | -56,5 (-63,2, -48,5) | 39 |
| IgG NFL | 0,39 (0,34, 0,46) | -60,7 (-66,4, -54,1) | 45 |
| IgG NCt | 0,25 (0,18, 0,34) | -75,4 (-82,0, -66,3) | 26 |
| IgG RBD | 0,56 (0,47, 0,67) | -44,2 (-53,5, -33,1) | 25 |
| IgG S | 0,36 (0,29, 0,45) | -63,9 (-70,8, -55,4) | 30 |
| IgG S2 | 0,58 (0,51, 0,66) | -42,2 (-49,3, -34,1) | 33 |

| Table S4. Spearman correlations for RBD antigen. All participants, n=1,076. Darker red=stronger association | | | | | | | | | | | | | | | |
| --- | --- | --- | --- | --- | --- | --- | --- | --- | --- | --- | --- | --- | --- | --- | --- |
|  | IgM Wuhan | IgM Alpha | IgM Beta | IgM Gamma | IgM Delta | IgA Wuhan | IgA Alpha | IgA Beta | IgA Gamma | IgA Delta | IgG Wuhan | IgG Alpha | IgG Beta | IgG Gamma | IgG Delta |
| IgM Wuhan | 1,000 |  |  |  |  |  |  |  |  |  |  |  |  |  |  |
| IgM Alpha | 0,852 | 1,000 |  |  |  |  |  |  |  |  |  |  |  |  |  |
| IgM Beta | 0,503 | 0,586 | 1,000 |  |  |  |  |  |  |  |  |  |  |  |  |
| IgM Gamma | 0,644 | 0,791 | 0,698 | 1,000 |  |  |  |  |  |  |  |  |  |  |  |
| IgM Delta | 0,819 | 0,864 | 0,564 | 0,729 | 1,000 |  |  |  |  |  |  |  |  |  |  |
| IgA Wuhan | 0,430 | 0,400 | 0,165 | 0,255 | 0,452 | 1,000 |  |  |  |  |  |  |  |  |  |
| IgA Alpha | 0,424 | 0,407 | 0,176 | 0,262 | 0,454 | 0,973 | 1,000 |  |  |  |  |  |  |  |  |
| IgA Beta | 0,372 | 0,378 | 0,209 | 0,275 | 0,420 | 0,858 | 0,879 | 1,000 |  |  |  |  |  |  |  |
| IgA Gamma | 0,397 | 0,387 | 0,196 | 0,287 | 0,439 | 0,902 | 0,924 | 0,937 | 1,000 |  |  |  |  |  |  |
| IgA Delta | 0,416 | 0,400 | 0,181 | 0,265 | 0,462 | 0,958 | 0,948 | 0,891 | 0,920 | 1,000 |  |  |  |  |  |
| IgG Wuhan | 0,420 | 0,371 | 0,131 | 0,252 | 0,439 | 0,764 | 0,735 | 0,642 | 0,686 | 0,736 | 1,000 |  |  |  |  |
| IgG Alpha | 0,412 | 0,366 | 0,125 | 0,243 | 0,430 | 0,754 | 0,726 | 0,632 | 0,674 | 0,725 | 0,994 | 1,000 |  |  |  |
| IgG Beta | 0,395 | 0,349 | 0,124 | 0,238 | 0,414 | 0,744 | 0,718 | 0,637 | 0,676 | 0,718 | 0,984 | 0,984 | 1,000 |  |  |
| IgG Gamma | 0,408 | 0,357 | 0,126 | 0,248 | 0,429 | 0,749 | 0,721 | 0,638 | 0,682 | 0,724 | 0,987 | 0,987 | 0,993 | 1,000 |  |
| IgG Delta | 0,417 | 0,366 | 0,127 | 0,250 | 0,436 | 0,760 | 0,729 | 0,639 | 0,685 | 0,735 | 0,993 | 0,990 | 0,987 | 0,990 | 1,000 |

| Table S5. Cross tabulation between serostatus to RBD of Wuhan variant with the RBD of Alpha, Beta Gamma and Delta variants. | | | |
| --- | --- | --- | --- |
|  | Wuhan RBD | | |
|  | negative | undetermined | positive |
| Alpha RBD |  |  |  |
| Negative | 87 (95.6%) | 18 (75%) | 8 (0.8%) |
| Indeterminate | 3 (3.3%) | 5 (20.8%) | 33 (3.4%) |
| Positive | 1 (1.1%) | 1 (4.2%) | 920 (95.7%) |
| Beta RBD |  |  |  |
| Negative | 85 (93.4%) | 22 (91.7%) | 126 (13.1%) |
| Indeterminate | 4 (4.4%) | 2 (8.3%) | 85 (8.8%) |
| Positive | 2 (2.2%) | 0 (0%) | 750 (78.1%) |
| Gamma RBD |  |  |  |
| Negative | 87 (95.6%) | 4 (4.4%) | 0 (0%) |
| Indeterminate | 24 (100%) | 0 (0%) | 0 (0%) |
| Positive | 53 (5.5%) | 45 (4.7%) | 863 (89.8%) |
| Delta RBD |  |  |  |
| Negative | 85 (93.4%) | 4 (4.4%) | 2 (2.2%) |
| Indeterminate | 21 (87.5%) | 2 (8.3%) | 1 (4.2%) |
| Positive | 25 (2.6%) | 45 (4.7%) | 891 (92.7%) |

| Table S6. Characteristics of non-responders (seronagetive or with an undetermined status) to vaccination. | | | | | | | | | |
| --- | --- | --- | --- | --- | --- | --- | --- | --- | --- |
|  | Serostatus | 1st vaccine | 2nd vaccine | age | sex | BMI status | smoking | time since 1st dose | time since 2nd dose |
| Incompletely vaccinated | | | | | | | | | |
| 1 | Negative | Vaxzevria |  | 62 | Male | Obese | Non-smoker | 77 |  |
| 2 | Negative | Comirnaty |  | 49 | Female | Obese | Non-smoker | 7 |  |
| 3 | Negative | Comirnaty |  | 46 | Female | Obese | Ex-smoker | 6 |  |
| 4 | Negative | Comirnaty |  | 47 | Male | Overweight | Non-smoker | 5 |  |
| 5 | Negative | Vaxzevria |  | 61 | Male | Obese | Ex-smoker | 82 |  |
| 6 | Negative | Comirnaty |  | 47 | Female | Obese | Ex-smoker | 2 |  |
| 7 | Negative | Vaxzevria |  | 65 | Male | Obese | Ex-smoker | 83 |  |
| 8 | Negative | Comirnaty |  | 46 | Female | Overweight | Smoker | 1 |  |
| 9 | Negative | Comirnaty |  | 49 | Female | Obese | Smoker | 7 |  |
| 10 | Negative | Comirnaty |  | 46 | Male | Overweight | Ex-smoker | 7 |  |
| 11 | Indeterminate | Comirnaty |  | 46 | Male | Overweight | Non-smoker | 3 |  |
| 12 | Indeterminate | Vaxzevria |  | 60 | Male | Underweight / Normal | Smoker | 65 |  |
| 13 | Indeterminate | Comirnaty |  | 49 | Female | Underweight / Normal | Non-smoker | 7 |  |
| 14 | Indeterminate | Vaxzevria |  | 65 | Male | Underweight / Normal | Non-smoker | 76 |  |
| 15 | Indeterminate | Vaxzevria |  | 64 | Female | Obese | Non-smoker | 73 |  |
| Completely vaccinated | | | | | | | | | |
| 16 | Negative | Janssen COVID-19 vaccine |  | 61 | Female | Overweight | Non-smoker | 9 |  |
| 17 | Negative | Vaxzevria | Vaxzevria | 59 | Female | Obese | Non-smoker | 77 | 5 |
| 18 | Indeterminate | Vaxzevria | Vaxzevria | 64 | Male | Overweight | Ex-smoker | 83 | 1 |
| 19 | Indeterminate | Vaxzevria | Vaxzevria | 69 | Male | Overweight | Smoker | 68 | 4 |
| 20 | Indeterminate | Vaxzevria | Vaxzevria | 69 | Male | Obese | No | 70 | 2 |

**Supplementary figures**

Figure S1. Dates and density of positive viral detection tests, sampling in 2020 (1st serological assessment) and 2021 (2nd serological assessment) and receipt of 1st vaccine dose in the study population (n=1,076).

Figure S2. Venn diagram illustrating overlap between sustainer groups of IgA or IgG antibodies against nucleoprotein and spike antigens, among all infected unvaccinated participants (n=64).

Figure S3. Differences in IgG antibody responses against RBD between Wuhan , Alpha, Beta, Gamma and Delta variant among vaccinated people. All differences were statistically significant apart from Delta vs Wuhan (p=0.861) and Alpha vs Wuhan (p=0.051)

**Figure S4.** Generalized additive models for associations of days since vaccination with antibody responses to the six isotype-antigen combinations in infected (red) and naïve (blue) participants after first or second dose in people vaccinated by Vaxzevria (a), Comirnaty (b) or Spikevax (c). Fitted lines after adjustment for participant’s age. Plus symbols (+) represent measured responses for a specific participant

**Figure S5.** Differences in antibody responses by infection and/or vaccination and number of doses in people vaccinated with Comirnaty (a), Spikevarx (b), Vaxzevria (c) of Janssen COVID-19 vaccine (d).

**Figure S6**. Differences in IgM responses by infection and/or vaccination and number of doses. Table S8 presents corresponding p-values.
